# The Subset Sum game^☆^

**DOI:** 10.1016/j.ejor.2013.08.047

**Published:** 2014-03-16

**Authors:** Andreas Darmann, Gaia Nicosia, Ulrich Pferschy, Joachim Schauer

**Affiliations:** a University of Graz, Institute of Public Economics, Universitaetsstr. 15, A-8010 Graz, Austria; b Dipartimento di Ingegneria, Università degli Studi “Roma Tre”, via della Vasca Navale 79, 00146 Rome, Italy; c University of Graz, Department of Statistics and Operations Research, Universitaetsstr. 15, A-8010 Graz, Austria

**Keywords:** Subset Sum problem, Multi-agent optimization, Performance analysis, Game theory

## Abstract

In this work we address a game theoretic variant of the Subset Sum problem, in which two decision makers (agents/players) compete for the usage of a common resource represented by a knapsack capacity. Each agent owns a set of integer weighted items and wants to maximize the total weight of its own items included in the knapsack. The solution is built as follows: Each agent, in turn, selects one of its items (not previously selected) and includes it in the knapsack if there is enough capacity. The process ends when the remaining capacity is too small for including any item left.

We look at the problem from a single agent point of view and show that finding an optimal sequence of items to select is an NP-hard problem. Therefore we propose two natural heuristic strategies and analyze their worst-case performance when (1) the opponent is able to play optimally and (2) the opponent adopts a greedy strategy.

From a centralized perspective we observe that some known results on the approximation of the classical Subset Sum can be effectively adapted to the multi-agent version of the problem.

## Highlights

• A game theoretic version of the Subset Sum problem is considered.
• Two agents take turns to fill a shared knapsack with their items.
• Natural heuristic strategies are proposed and analyzed from a worst-case perspective.

## 1 Introduction

In the computer science literature of the last two decades several classical combinatorial optimization problems have been revisited in a game theoretic setting where multiple deciders take over the role of a single decision maker. This new field of research is receiving growing attention and led to the emergence of *algorithmic game theory* (Nisan, Roughgarden, Tardos, & Vazirani, 2007).

In this context we address the problem of two agents competing for a shared resource. It can be described as a game theoretic variant of the classical *Subset Sum problem*, in which there are two players, or agents, called *P*_*a*_ and *P*_*b*_, and a given amount *c* of a shared resource. Each agent owns a set of items with non-negative weights and knows the other agent’s item set, i.e. there is perfect information.

The *Subset Sum game* works as follows: Starting with *P*_*a*_, the agents take turns to select exactly one of their items which was not selected before. The total weight of all selected items must not exceed the capacity *c* at any time. The aim of the game is, for each agent, to select a subset of its items with maximum total weight. This problem is new and has not been studied in the literature before. In the following we point out some possible application scenarios.

### 1.1 Motivation

The Subset Sum game is an interesting theoretical problem in its own right as a game theoretic version of the most basic combinatorial optimization problem. Moreover, one can find applications in all scenarios where a limited resource has to be allocated to different and possibly selfish users.

In the general setting of a single-machine scheduling problem two agents may compete for the processing time and want to maximize their utilization of the machine within a given planning horizon by scheduling some of their jobs. To decide in a fair way which jobs to accept for processing, a round-robin mechanism is often the method of choice (see for instance Agnetis, Pacciarelli, & Pacifici, 2007; Auletta, De Prisco, Penna, & Persiano, 2009). It remains for the agents to decide which jobs to submit.

In many communication networks there is the need to allocate bandwidth based on user requests (using, for example, the RSVP-TE protocol Awduche et al., 2001). Consider a single time unit in which two agents (users) have a number of different bandwidth requirements, corresponding to different applications, to be allocated. In a simplified scenario the users are asked to submit their requests with a round-robin discipline and want to maximize their allocated requests. More details on this application domain and related context can be found in the next section.

In a business setting a highly specialized producer, say a much sought-after dress maker, may produce creations for two designers. From a long term business perspective, the producer wants to retain both designers as customers, however, the production capacity up to the given deadline (the fashion show) is limited. Thus, the producer accepts orders alternating between the two designers as long as the production capacity suffices to fulfill the current set of orders. It remains to decide for the designers which creations to submit to this allocation mechanism and which to produce elsewhere with a less desirable producer.

### 1.2 Related works

Several problems strictly related to the Subset Sum game have been considered in the literature. In particular, due to its simple structure, the {0, 1}-Knapsack problem was frequently considered in a game-theoretic context and will be briefly discussed in Section 5. Recently, (Marini, Nicosia, Pacifici, & Pferschy, 2013; Nicosia, Pacifici, & Pferschy, 2011) considered a knapsack-type scenario with unitary weights in which the decision process is performed in rounds and managed by a central decision mechanism (arbitrator). In every round each of the two agents selects exactly one of its items and submits the item for possible inclusion in the knapsack, then the arbitrator chooses one of the items as “winner” of the round. The winning item is permanently included in the knapsack. The process goes on as long as there is enough capacity. A related problem where jobs are submitted in rounds for selection to be processed on a single machine was recently considered in Agnetis, Nicosia, Pacifici, and Pferschy (in press).

In the so called *Knapsack Sharing* problem studied by several authors (see for instance Fujimoto & Yamada, 2006; Hifi, M’Hallab, & Sadfi, 2005), a single objective function balancing the profits among the agents is considered in a centralized perspective. Another interesting game, based on the maximum {0, 1}-Knapsack, interpreted as a special on-line problem, is addressed in Liberatore (2000) where a two person zero-sum game, called *Knapsack Game*, is considered. Knapsack problems are also addressed in the context of auctions, see for example (Aggarwal & Hartline, 2006; Brotcorne, Hanafi, & Mansi, 2009).

Kindred problems are the so-called *Bin Packing Games*. There, one considers *k* agents representing bins and another *n* agents representing items of given size. The value function of a coalition of bins and items is the maximum total size of items in the coalition that can be packed into the bins of the coalition. This class of problems was introduced in Faigle and Kern (1993, 1998) where numerous results are provided. The *Selfish Bin Packing* is another interesting game theoretic variant, where each item is controlled by a selfish agent who pays a cost proportional to the ratio between its own item size and the total weight of the items packed in the same bin. In Epstein and Kleiman (2011), the authors study different equilibria and the associated quality measures, namely Price of Anarchy and Price of Stability.^1^

A significant application closely related to our problem is the so called *Admission Control* problem (ACP) which, in a wide sense, refers to the design of mechanisms for managing traffic requests in communication systems (for a comprehensive survey, see for instance (Ahmed, 2005; Ghaderi & Boutaba, 2006)). In bandwidth-managed networks, it is required to evaluate (i) if bandwidth is available to service a new potential user and (ii) the QoS (quality of service) that can be provided to this user. Only if bandwidth is available, the new user is admitted. Clearly, new users can be viewed as selfish users competing for network bandwidth, i.e., resource capacity. In this context, game theory techniques have been used to design management protocols to monitor, control, and enforce the use of shared resources and services in networks. For instance, in Yolken and Bambos (2008) the authors propose pricing schemes influencing users in their decision to take part or not in a wireless channel. The equilibria induced by these schemes and their performance are evaluated showing their potential to produce high quality outcomes in an incentive-compatible way. Analogously, the issue of designing resource allocation mechanisms that produce efficient throughput and congestion allocations despite the selfish users’ behavior is discussed in Shenker (1995).

### 1.3 Our contributions

The above mentioned works follow two established approaches in classical and algorithmic game theory and focus on (i) finding equilibria, i.e., solutions where each agent obtains no benefit by moving away from the current solution, and/or (ii) designing mechanisms, i.e. protocols leading the (selfish) agents to solutions which are either globally optimal or follow some intuitive, easy to compute rule. The problem we address in this paper is indeed a multi-deciders version of the well known Subset Sum problem where two agents compete for the capacity of a common knapsack in presence of a very simple mechanism (round robin). However, we adopt a different perspective here, namely we look at the problem from the point of view of one agent and seek strategies optimizing her payoff depending on the behavior of the other agent.

In this paper we show that it is NP-hard to compute an optimal strategy for one agent by a simple reduction from the standard Subset Sum problem. Hence, we introduce heuristic approaches and analyze two very natural strategies based on a greedy concept which would be intuitive rules of thumb for any practical game scenario (Section 2.2).

The first strategy is the pure greedy algorithm, which maximizes in each round the weight of the selected item. The second strategy is an extension which tries to take the subsequent round into account in the decision. Assuming that one agent adopts the proposed strategy, we analyze the performances when (i) the opponent is able to play optimally and (ii) the opponent also follows the greedy strategy. In particular, we are able to show that the first heuristic has a performance ratio of 1/2 both against an optimal opponent and against a greedy opponent (Section 3.1), while the second proposed algorithm has a performance ratio of 2/3 against the optimal opponent (see Section 3.2). Moreover, we show that a natural generalization of these greedy based strategies invoking a farther reaching consideration of future rounds does not allow a further improvement of the 2/3 performance ratio (Section 3.3).

Furthermore, we observe that from a centralized perspective, some known results on the approximation of the classical Subset Sum can be effectively adapted to the multi-agent version of the problem (Section 4).

After showing in Section 5 that two natural extensions of the proposed algorithms to a Knapsack Game problem do not provide a bounded performance ratio, we conclude with a few directions for future research (Section 6).

### 1.4 Formal problem setting

Let *P*_*a*_ and *P*_*b*_ indicate the two agents. Agent *P*_*x*_ owns a set *N*_*x*_ of *n*_*x*_ items, where item *i* of agent *P*_*x*_ has a non-negative weight *x*_*i*_, with *x* = *a*, *b*. We assume that *N*_*a*_ ∩ *N*_*b*_ = ∅ and that there is perfect information, so agents know each other’s item sets.

The game can be seen as a sequence of *rounds*, where in each round *P*_*a*_ selects an item from *N*_*a*_ followed by the selection of an item from *N*_*b*_ by agent *P*_*b*_. The total weight of all selected items must not exceed the capacity *c* at any time. If at any point of the game one agent is unable to select any more items because the remaining capacity is too small, the agent just remains idle and the other agent can continue to select items.

It is easy to show that the associated decision problem, namely whether both agents can reach a certain total weight, is NP-complete.

Subset Sum Game Decision (SSGD): Given *a*_*j*_ and *b*_*j*_, *j* = 1, … , *n*, and two positive values *Q*_*a*_ and *Q*_*b*_. Is there an outcome of the Subset Sum game such that *P*_*a*_ gains a total weight ⩾ *Q*_*a*_ and *P*_*b*_ gains ⩾ *Q*_*b*_?

Proposition 1 *Problem SSGD is* NP*-complete.*

Proof Reduction from the Subset Sum problem (SSP): Given *n* integer numbers *w*_1_, … , *w*_*n*_ and a value *W*, is there a subset *S* of items with total weight equal to *W*?

To answer the decision version of SSP consider the following instance *I* of SSGD: Agent *P*_*a*_ tries to solve SSP, i.e. *a*_*i*_ = *w*_*i*_ for *i* = 1, … , *n*. Agent *P*_*b*_ is negligible with *b*_*i*_ = *ε* for all *i* and *c* = *W* + *nε*, where *ε* < 1/*n*. Set *Q*_*a*_ = *W* and *Q*_*b*_ = *ε*. It is easy to see that the strategies pursued by the two agents do not matter at all since *P*_*b*_ always can select all its items while *P*_*a*_ never can exploit the capacity *c* − *W* < 1. *P*_*a*_ can reach *Q*_*a*_ = *W* iff *SSP* is a YES-instance. □

## 2 Strategies for one agent

For notational convenience, we address the problem from the point of view of agent *P*_*a*_. For agent *P*_*b*_, the perspective is completely the same after subtracting from *c* the weight of the item selected by *P*_*a*_ in the first round.

In this paper, with a slight abuse of terminology, a *strategy*^2^ *S* of an agent indicates a rule, or an algorithm, that specifies which item to select in any round depending on the capacity and the sets of selected and still available items of both agents. Since the outcome of the game depends on the strategies employed by the two agents, if *P*_*a*_ follows a strategy *S* and *P*_*b*_ a strategy *Z*, then we denote the total weights obtained by agent *P*_*a*_ and *P*_*b*_ as *A*_*SZ*_ and *B*_*SZ*_, respectively. Clearly, for every pair of feasible strategies *S* and *Z*, *A*_*SZ*_ + *B*_*SZ*_ ⩽ *c*.

### 2.1 Optimal strategy

In general, the optimal strategy of an agent depends not only on the outcome of previous rounds but also on the future decisions of the other agent. If the strategy of *P*_*b*_ is not known, there is no way for *P*_*a*_ to always make optimal decisions. Else, if agent *P*_*a*_ knows the strategy *Z* of agent *P*_*b*_, it can compute an *optimal strategy O*, maximizing the total weight of the selected items. This can be done by modeling the Subset Sum game as a *game in extensive form* and representing it by a *game tree* as it is usually done in game theory (see e.g. Osborne, 2004, Section 5).

A game tree represents all possible decisions of both agents in sequential form. Each node of the tree corresponds to the decision of an agent in a certain round, such that every possible outcome of this decision is represented by a child node. Thus, the root of the tree (i.e. a node in level 1) corresponds to the decision of *P*_*a*_ in the first round and has *n*_*a*_ child nodes, one for each possible item selected by *P*_*a*_. Each such child node (i.e. a node in level 2) represents the decision of *P*_*b*_ in the first round. Clearly, feasible selections are those where the total weight reached at the current node does not exceed the capacity *c*.

Considering an arbitrary node in level ℓ, ℓ ⩾ 2, in the tree, one could easily determine all previous decisions by moving upwards along the unique path to the root of the tree. Thus, the remaining capacity and the set of not yet selected items of the current agent (*P*_*a*_ if ℓ ≡ 1 mod 2, *P*_*b*_ otherwise) are known and one can establish which items could still be selected at this node. Each of them gives rise to a child node in level ℓ + 1. It is convenient to assume that an agent selects an artificial item of weight 0 if it cannot select any other item, but the other agent still has items to select. Every leaf of this game tree describes a feasible outcome of the game and yields a pair of total weights (*A*, *B*) obtained by the two agents.

Now an *optimal strategy* can be determined for both agents by settling all decisions by *backward induction*. This means that for each node, whose child nodes are all leaves, the associated agent can reach a final decision by simply choosing the best of all child nodes w.r.t. their allocated total weight. Then these leaf nodes can be deleted and the pair of gained weights of the chosen leaf is moved to its parent node. In this way, we can move upwards in the tree towards the root and settle all decisions along the way.

If an agent, say *P*_*b*_, follows a certain strategy *S*, then *P*_*b*_ will execute a certain decision in each of its nodes. Thus, each of its nodes only has one child node. *P*_*a*_ can determine an optimal answer against strategy *S* by following the same procedure as above.

Unfortunately, this procedure cannot be easily used in practice due to the exponential number of nodes in the game tree. In particular, it can be easily concluded from the proof of Proposition 1, that it is NP-hard to compute the optimal strategy for an agent against a given strategy of the other agent. To escape this intractability, it is a reasonable approach to make use of heuristic algorithms as strategies (see in Sections 2.2 and 3).

In the terminology of game theory an optimal strategy as described above is by definition a *Nash equilibrium* and also a so-called *subgame perfect equilibrium* (a slightly stronger property), since the decisions made in the above backward induction procedure are also optimal for every subtree (see Osborne (2004, Section 5) for more details). Clearly, the optimal strategy and hence the equilibrium of the game is not unique, since there may well be several different leafs of the game tree yielding the same weights for both agents.

### 2.2 Heuristic algorithms

Because it is NP-hard to compute an optimal strategy, we will consider heuristic strategies for the agents. A simple greedy algorithm is a very natural choice for the Subset Sum game. In this case an agent simply selects in every round the item with largest weight that does not violate the capacity constraint. In this way, the capacity available for the other agent is (at least locally) minimized, which seems to be an intuitively appealing approach. In Section 3.1 it is shown that such a greedy algorithm may reach only half of the weight obtained by an optimal strategy but cannot do worse than that. For sake of completeness we also give a formal description in Algorithm 1.

Algorithm 1 Greedy *G*

**Table:** 

$N≔N_{a}\text{;}\;\overset{¯}{c}≔c$;
**while**$\min\{a_{j}|j\in N\}⩽\overset{¯}{c}$**do**
$a_{\max}≔\max\{a_{j}|a_{j}⩽\overset{¯}{c}\text{,}j\in N\}$ attained for $\overset{˜}{}$;
select item $\overset{˜}{}\text{;}N≔N-\{\overset{˜}{}\}$;
selection of an item *b*′ by *P*_*b*_;
$\overset{¯}{c}≔\overset{¯}{c}-a_{\overset{˜}{}}-b^{\prime }$
**end while**

An effective improvement over this simple greedy mechanism is the following Look-ahead Greedy algorithm *L*, which tries to avoid the shortsightedness of Greedy at least to some extent. Motivated by the worst-case example given in Section 3.1 (proof of Theorem 5) it considers in every round all *feasible pairs* of items and picks the pair with highest total weight. The larger item of this pair is then selected in the current round. In this context a pair of items is feasible if *P*_*a*_ can be sure to be able to select the two items in the current and the subsequent round, no matter what *P*_*b*_ does in the current round, i.e. even if *P*_*b*_ selects the largest item that fits. A formal description is given in Algorithm 2. To avoid tedious special cases we assume that there are always sufficient items available for *P*_*a*_ to consider a pair of items in every round. This can be achieved by adding dummy items with weight 0.

Algorithm 2 Look-ahead Greedy *L*

**Table:** 

$N≔N_{a}\text{;}\overset{¯}{c}≔c$;
**while**$\min\{a_{j}|j\in N\}⩽\overset{¯}{c}$**do**
*p*_max_≔0;
**for** every pair (*i*, *j*) in *N* with *a*_*i*_ ⩾ *a*_*j*_**do**
$b_{\max}≔\max\{b_{\ell }|b_{\ell }⩽\overset{¯}{c}-a_{i}\}$
**if**$a_{i}+b_{\max}+a_{j}⩽\overset{¯}{c}$**then**
*p*_max_≔max{*p*_max_, *a*_*i*_ + *a*_*j*_}
**end if**
**end for**
let *p*_max_ be attained for $\left(\overset{˜}{ı}\text{,}\overset{˜}{}\right)$;
select item $\overset{˜}{ı}\text{;}N≔N-\{\overset{˜}{ı}\}$;
selection of an item *b*′ by *P*_*b*_;
$\overset{¯}{c}≔\overset{¯}{c}-a_{\overset{˜}{ı}}-b^{\prime }$
**end while**

It should be noted that algorithm Look-ahead Greedy may also select an item different from $\overset{˜}{}$ in the next round if a different pair of items without $\overset{˜}{}$ turns out to be the best choice in the next round or if *P*_*b*_ does not select the maximal possible weight *b*_max_ in the current round.

A natural generalization of Look-ahead Greedy looks even farther into the future and considers more than two items as a look ahead. The resulting *k*-Look-ahead Greedy algorithm (*k*-L) determines, in each round, the best combination of *k* items for *P*_*a*_ that cannot be “blocked” by the opponent *P*_*b*_. Then the largest item of this *k*-tuple is selected in this round. Clearly, the above algorithm *L* arises as the special case of 2-L. Note that by definition of this algorithm, any possibility by *P*_*b*_ to block the *k*-tuple considered by *P*_*a*_ is taken into account. This “blocking strategy” of *P*_*b*_ may be quite different from the greedy strategy which always puts *b*_max_ against 2-L.

While it is easy to see that Look-ahead Greedy may perform better than Greedy (see e.g. Example 3), and also 3-Look-ahead Greedy may perform better than 2-Look-ahead Greedy (e.g. in Example 5), we can show that it may also be the other way round (see e.g. Example 1). In conclusion, there is no strict dominance between the different extensions of the Greedy strategy.

Example 1 Consider the following instance of the Subset Sum game with capacity 12 + 5*δ* where *δ* ≪ *ε*.

**Table:** 

Item	1	2	3	4	5	6	7	8
*N*_*a*_	10	4 − *ε*	4 − *ε*	4 − *ε*	$\frac{1}{2}$	$\frac{1}{2}$	$\frac{1}{2}$	$\frac{1}{2}$
*N*_*b*_	*δ*	*δ*	*δ*	*δ*	*δ*

Observe that the items in *N*_*b*_ can all be packed and can be neglected in the selection of *P*_*a*_. 3-Look-ahead Greedy identifies the three items with weight 4 − *ε* as the best triple and selects one of them. In round 2, only the remaining two items of this triple can be packed, and the algorithm continues to gain a total weight of 12 − 3*ε*.

An optimal selection of *P*_*a*_ would start with item 1 and continues to pack all four items of weight $\frac{1}{2}$ ending up with a total weight of 12. Note that Greedy and 2-Look-ahead Greedy both pick this optimal strategy.

A simple variation of this example where the three items of weight 4 − *ε* are replaced by two items of weight 6 − *ε* shows by an analogous reasoning that 2-Look-ahead Greedy may be stuck with 12 − 2*ε* while the optimal solution identified by Greedy obtains 12.

## 3 Performance analysis

To analyze the performance of the heuristics for the Subset Sum game we follow a worst-case perspective taking agent *P*_*a*_’s point of view. As in the performance analysis of classical approximation algorithms, we consider the worst solution scenario over all possible instances, i.e. sets of input data, and compare the solution value derived by a heuristic with the solution value attained by the optimal strategy.

Differently from classical optimization problems we also have to include the strategy of agent *P*_*b*_ in our analysis. In the following we define as *performance bound ρ*_*HS*_ ∈ [0, 1] a bound on the ratio between the solution value of a heuristic *H* and the solution value of an optimal strategy, denoted by *O*, for agent *P*_*a*_ both playing against a specified strategy *S* of the adversary agent *P*_*b*_, i.e.

(1) $$\rho_{\mathrm{HS}}⩽\frac{A_{\mathrm{HS}}}{A_{\mathrm{OS}}}\;\mathrm{for}\;\mathrm{all}\;\mathrm{inputs}\text{.}$$

As usual, we call a performance bound *ρ*_*HS*_ *tight*, if no larger value than *ρ*_*HS*_ exists which fulfills (1).

Assuming that *P*_*b*_ does not act in a completely arbitrary or self destructive way, the most plausible strategy *S* of *P*_*b*_ is the optimal response. That is, knowing the strategy *H* of *P*_*a*_, *P*_*b*_ maximizes its total weight (cf. Section 2.1) neither trying to help nor harm *P*_*a*_. By maximizing its own total weight, the capacity remaining to be utilized by *P*_*a*_ is automatically minimized, which fits well together with the notion of a worst-case analysis. However, note that an optimal strategy of *P*_*b*_ does not necessarily yield the worst possible outcome for a strategy of *P*_*a*_. This can be seen—after exchanging the roles of *P*_*a*_ and *P*_*b*_—from Example 4, where the switching from the optimal to the Greedy strategy of one agent generates a worse outcome for the optimal strategy of the other agent.

To avoid clumsy notation we assume w.l.o.g. that the items of both agents are numbered in the order they are selected during the game, i.e. agent *P*_*a*_ selects *a*_*j*_ in round *j*. Items not selected are numbered arbitrarily with indices higher than the selected items.

The following proposition will be useful in the proofs below.

Proposition 2 *It can always be assumed that an optimal strategy of P*_*b*_ *selects items in nonincreasing order of weights against the* Greedy *strategy of P*_*a*_*.*

Proof Assume contrary to the statement that there exists a selected item *b*_*j*_ such that *b*_*j*−1_ < *b*_*j*_, *j* ⩾ 2. Clearly, there must be

(2) $$\overset{j}{\underset{k=1}{\sum }}b_{k}⩽c-\overset{j}{\underset{k=1}{\sum }}a_{k}\Leftrightarrow a_{j}⩽c-\overset{j-1}{\underset{k=1}{\sum }}a_{k}-\overset{j}{\underset{k=1}{\sum }}b_{k}\text{.}$$

Since *a*_*j*_ was computed as the maximum over all remaining items with capacity at most $c-\sum_{k=1}^{j-1}a_{k}-\sum_{k=1}^{j-1}b_{k}$, and since *a*_*j*_ also fulfills the stricter condition (2), it follows that *a*_*j*_ is also the maximum over all remaining items with the intermediate capacity at most $c-\sum_{k=1}^{j-1}a_{k}-\sum_{k=1}^{j-2}b_{k}-b_{j}$. Therefore, the Greedy algorithm of *P*_*a*_ does not change its selection in round *j* even if *P*_*b*_ selects *b*_*j*_ in round *j* − 1 and *b*_*j*−1_ in round *j*. □

Note that Proposition 2 does not hold for the Look-ahead Greedy strategy, as shown in the following example:

Example 2 Consider the following instance I of the Subset Sum game with capacity *c* = 19 + 3*ε*.

**Table:** 

Item	1	2	3	4
*N*_*a*_	6	6	3 + *ε*	3 + *ε*
*N*_*b*_	5 + 4*ε*	*5*	2	3*ε*

In the first round *P*_*a*_ computes the largest pair consisting of items 1 and 2 and thus chooses item 1 with weight 6 leaving the residual capacity $\overset{¯}{c}=13+3\varepsilon$. *P*_*b*_ has four possibilities to react. The resulting games are listed as columns in Table 1.

In order to achieve the maximum total weight for herself, *P*_*b*_ has to choose first the item with weight 2 and then the one with weight 5. In some sense *P*_*b*_ can “threaten” to use its largest item 1 in the next round and thereby forces *P*_*a*_ to choose the larger item with weight 6 instead of items 3 and 4. Thereby Look-ahead Greedy leaves room for the final item 3*ε* of *P*_*b*_. If *P*_*b*_ chose items in decreasing order of their weights, this would permit a better choice for *P*_*a*_ and would reduce the total weight attained by *P*_*b*_.

By going through all possible solutions it can be checked that in the above example the strategy of *P*_*a*_ is optimal.

We summarize the results of Example 2 in the following proposition.

Proposition 3 *The optimal strategy of agent P*_*b*_ *against L*ook-ahead *G*reedy *or against an optimal strategy of agent P*_*a*_ *may select items in a non-monotone order of weights.* □

### 3.1 Performance of the Greedy Algorithm

The following technical lemma will be used to prove the performance bound of Greedy both against an optimal and against a greedy strategy of *P*_*b*_.

Lemma 4 *Let agent P*_*a*_ *use the* Greedy *strategy G and let S be the strategy used by P*_*b*_*. Assume that* Greedy *is able to select the largest j* − *1 items of N*_*a*_ *and fails to pack the jth largest item in round j against S. If S* ∈ {*O, G*} *then*

$$A_{\mathrm{OS}}⩽c-\overset{j-1}{\underset{k=1}{\sum }}b_{k}\text{,}$$

*where b*_*1*_*,* … *,* *b*_*j*−*1*_ *are the items selected by P*_*b*_ *in the first j* − *1 rounds following strategy S against the* Greedy *strategy G of P*_*a*_*.*

Proof We assume *j* ⩾ 2, since the statement is trivial for *j* = 1. Since *A*_*OS*_ ⩽ *c* − *B*_*OS*_, it is sufficient to show that for *S* ∈ {*O*, *G*} we have $B_{\mathrm{OS}}⩾\sum_{k=1}^{j-1}b_{k}$.

Let *S* = *O*, i.e., *P*_*b*_ uses its optimal strategy. Then, even an optimal strategy of *P*_*a*_ cannot avoid that *P*_*b*_ reaches a total weight *B*_*OO*_ of at least $\sum_{k=1}^{j-1}b_{k}$, which *P*_*b*_ managed to achieve after *j* − 1 rounds even against the largest *j* − 1 items of *N*_*a*_.

Let *S* = *G*. Assume that $B_{\mathrm{OG}}<\sum_{k=1}^{j-1}b_{k}$. Let ${\overset{˜}{b}}_{1}\text{,}{\overset{˜}{b}}_{2}\text{,}\ldots \text{,}{\overset{˜}{b}}_{j-1}$ be the items selected in the first *j* − 1 rounds by *G* of *P*_*b*_ against *O* of *P*_*a*_. Then, in order to satisfy $B_{\mathrm{OG}}<\sum_{k=1}^{j-1}b_{k}$,

(3) $$\overset{j-1}{\underset{k=1}{\sum }}{\overset{˜}{b}}_{k}<\overset{j-1}{\underset{k=1}{\sum }}b_{k}$$

must hold. Since *P*_*b*_ uses the Greedy strategy in both scenarios (against *G* and against *O* of *P*_*a*_), there is a unique smallest index *i*, 1 ⩽ *i* ⩽ *j* − 1, such that ${\overset{˜}{b}}_{i}\;\neq \;b_{i}$. Note that because of *i* ⩽ *j* − 1, $c-\sum_{k=1}^{i}{\overset{˜}{a}}_{k}⩾c-\sum_{k=1}^{i}a_{k}$ holds, where ${\overset{˜}{a}}_{1}\text{,}\ldots \text{,}{\overset{˜}{a}}_{i}$ and *a*_1_, … , *a*_*i*_ denote the items selected by *P*_*a*_ according to *O* and *G*, respectively. If ${\overset{˜}{b}}_{i}<b_{i}$, then in round *i* it would have been possible for *P*_*b*_ to pack the larger item *b*_*i*_ against *O* of *P*_*a*_, which contradicts the fact that *P*_*b*_ uses the greedy strategy. Hence, ${\overset{˜}{b}}_{i}>b_{i}$ holds. However, because of (3) and the definition of *i*, $\sum_{k=i}^{j-1}b_{k}>\sum_{k=i}^{j-1}{\overset{˜}{b}}_{k}$ holds. This implies $\sum_{k=i}^{j-1}b_{k}>{\overset{˜}{b}}_{i}$, and hence, when playing against *G* of *P*_*a*_, *P*_*b*_ could have packed ${\overset{˜}{b}}_{i}>b_{i}$ in round *i*. Again, this contradicts the Greedy strategy. Therewith, $B_{\mathrm{OG}}⩾\sum_{k=1}^{j-1}b_{k}$ holds. □

Theorem 5 *The* Greedy *algorithm G has a tight performance bound of*

$$\rho_{\mathrm{GO}}=\frac{1}{2}\text{.}$$

Proof To show that $A_{\mathrm{GO}}⩾\frac{1}{2}A_{\mathrm{OO}}$ we assume *A*_*OO*_ > *A*_*GO*_ because otherwise *A*_*OO*_ = *A*_*GO*_ and we are done. By the greedy strategy *G* selects the largest *j* − 1 items of *N*_*a*_ and fails to pack the *j*th largest item with weight $\overset{˜}{a}$ in round *j* for some *j* ⩾ 2. It may continue to pack smaller items, but these can be neglected in our analysis. If $\sum_{k=1}^{j-1}a_{k}⩾\frac{1}{2}\;A_{\mathrm{OO}}$ we are done. Otherwise, for $\sum_{k=1}^{j-1}a_{k}<\frac{1}{2}\;A_{\mathrm{OO}}$ we apply a simple averaging argument to show

(4) $$\overset{˜}{a}⩽a_{j-1}⩽\frac{1}{j-1}\overset{j-1}{\underset{k=1}{\sum }}a_{k}<\frac{1}{j-1}\frac{1}{2}A_{\mathrm{OO}}⩽\frac{1}{2}A_{\mathrm{OO}}\text{.}$$

Since $\overset{˜}{a}$ could not be selected in round *j* there must be

(5) $$\overset{˜}{a}>c-\overset{j-1}{\underset{k=1}{\sum }}a_{k}-\overset{j-1}{\underset{k=1}{\sum }}b_{k}\;\Leftrightarrow \;\overset{j-1}{\underset{k=1}{\sum }}a_{k}>c-\overset{j-1}{\underset{k=1}{\sum }}b_{k}-\overset{˜}{a}\text{.}$$

Because of Lemma 4, $A_{\mathrm{OO}}⩽c-\sum_{k=1}^{j-1}b_{k}$ holds. Putting this inequality together with (5) and plugging in (4) we get

$$A_{\mathrm{OO}}-A_{\mathrm{GO}}⩽c-\overset{j-1}{\underset{k=1}{\sum }}b_{k}-\left(c-\overset{j-1}{\underset{k=1}{\sum }}b_{k}-\overset{˜}{a}\right)=\overset{˜}{a}⩽\frac{1}{2}A_{\mathrm{OO}}$$

and we have shown $\rho_{\mathrm{GO}}⩽\frac{1}{2}$.

The following Example 3 gives an instance with parameter *ε* > 0 where $\lim_{\varepsilon \to 0}\rho_{\mathrm{GO}}=\frac{1}{2}$ thus completing the proof of the theorem. □

Example 3 Consider the following instance with *c* = 1.

**Table:** 

Item	1	2	3
*N*_*a*_	$\frac{1}{2}$	$\frac{1}{2}-\varepsilon$	$\frac{1}{2}-\varepsilon$
*N*_*b*_	2*ε*	*ε*

In the first round *P*_*a*_ selects the largest item 1 and *P*_*b*_ chooses 2*ε*. Now *P*_*a*_ cannot select another item and $A_{\mathrm{GO}}=\frac{1}{2}$. An optimal strategy would select items 2 and 3 with a total weight of *A*_*OO*_ = 1 − 2*ε*.

Corollary 6 *Against the* Greedy *strategy G of P*_*b*_*, the* Greedy *strategy of P*_*a*_ *has a tight performance ratio of*

$$\rho_{\mathrm{GG}}=\frac{1}{2}\text{.}$$

Proof The proof is analogous to the one of Theorem 5 when *A*_*OO*_ (*A*_*GO*_) are replaced by *A*_*OG*_ (*A*_*GG*_). The tightness of the bound again can be concluded from the instance used in Example 3. □

It could be expected that the optimal strategy for *P*_*b*_ always yields a better solution against a suboptimal strategy of *P*_*a*_, such as *G*, than against an optimal strategy of *P*_*a*_. Clearly, if *P*_*a*_ consumes less capacity, there is more capacity left for *P*_*b*_ to utilize. Surprisingly, this is not always the case as shown by the following counterexample. It may be necessary for *P*_*b*_ to select a less attractive item in order to block another item of *P*_*a*_. However, if both agents pursue an optimal strategy, they might both benefit.

Example 4 Consider an instance with the following data and capacity *c* = 23.

**Table:** 

Item	1	2	3	4	5
*N*_*a*_	7	7	4	4	4
*N*_*b*_	10	5.5	5.5	0	0

If *P*_*a*_ follows the Greedy algorithm *G*, it first selects *a*_1_ = 7. If *P*_*b*_ selects an item with weight 5.5 in the first round, *P*_*a*_ could select the second item of weight 7 in the second round thus preventing *P*_*b*_ from picking a further item. Hence, *P*_*b*_ has to choose item *1* in the first round ending the game with *B*_*GO*_ = 10, while *P*_*a*_ can add an item of weight *4* in the second round obtaining *A*_*GO*_ = 11.

To find an optimal strategy we depict a partial decision tree below. Numbers in the circles refer to item weights while the leaves of the tree contain the weights obtained by both agents.

Clearly, *P*_*a*_ will start with an item of weight 4 because otherwise the above case applies again. If *P*_*b*_ chooses item 1 with weight 10 in the first round, *P*_*a*_ could select any item in the second round to prevent *P*_*b*_ from selecting any further items. Hence, *P*_*b*_ should select, in the first round, an item with weight 5.5. This allows *P*_*a*_ two choices: (1) it selects an item of weight 7, then *P*_*b*_ would continue with the other item of weight 5.5 and both agents are finished gaining a total weight of 11 each; (2) *P*_*a*_ selects another item of weight 4, then again *P*_*b*_ continues with the other item of weight 5.5 (since item 1 does not fit) and reaches a total weight of *B*_*OO*_ = 11 while *P*_*a*_ can enter into a next round to submit a third item of weight 4 yielding *A*_*OO*_ = 12 (circled outcome).

Thus, we have given an instance with *A*_*GO*_ < *A*_*OO*_ and *B*_*GO*_ < *B*_*OO*_.

While the optimal strategy of an agent may even suffer from a suboptimal strategy of its opponent, the following proposition says that for the Greedy strategy it is always better if the opponent deviates from an optimal response to a greedy response.

Proposition 7 Greedy *for P*_*a*_ *always benefits if P*_*b*_ *applies* Greedy *rather than an optimal strategy, that is*

$$A_{\mathrm{GG}}⩾A_{\mathrm{GO}}\text{.}$$

Proof We can assume *A*_*GG*_ ≠ *A*_*GO*_ because otherwise we are done. Let *a*_*k*_ (resp. ${\overset{˜}{a}}_{k}$) denote the item selected by *G* of agent *P*_*a*_ against *G* (resp. *O*) of *P*_*b*_ in round *k*, *k* ⩾ 1. Note that $a_{1}={\overset{˜}{a}}_{1}$ holds since *P*_*a*_ applies the greedy strategy in both scenarios. Thus, there must be an index *i* ⩾ 2 such that $a_{i}\;\neq \;{\overset{˜}{a}}_{i}$ and $a_{j}={\overset{˜}{a}}_{j}$ holds for *j* < *i*.

**Case 1**: ${\overset{˜}{a}}_{i}>a_{i}$. Then, in round *i* agent *P*_*a*_ was able to pack the larger item ${\overset{˜}{a}}_{i}$ against *O* of *P*_*b*_ but could not pack it against *G* of *P*_*b*_. Hence, $B_{\mathrm{GG}}>c-\sum_{k=1}^{i-1}a_{k}+{\overset{˜}{a}}_{i}$. On the other hand, since in the first *i* rounds *P*_*a*_ was able to pack the items ${\overset{˜}{a}}_{k}$, 1 ⩽ *k* ⩽ *i*, against *O* of *P*_*b*_, we must have
  $$B_{\mathrm{GO}}⩽c-\overset{i}{\underset{k=1}{\sum }}{\overset{˜}{a}}_{k}=c-\overset{i-1}{\underset{k=1}{\sum }}a_{k}+{\overset{˜}{a}}_{i}\text{.}$$
  I.e., *B*_*GG*_ > *B*_*GO*_ holds, in contradiction to the definition of *O*.
**Case 2**: ${\overset{˜}{a}}_{i}<a_{i}$. This means that in round *i*, agent *P*_*a*_ was able to pack the larger item *a*_*i*_ against *G* of *P*_*b*_ but could not pack it against *O* of *P*_*b*_. Thus, $B_{\mathrm{GO}}>c-\sum_{k=1}^{i-1}{\overset{˜}{a}}_{k}+a_{i}$, which settles the claim because it implies
  $$A_{\mathrm{GO}}<\overset{i-1}{\underset{k=1}{\sum }}{\overset{˜}{a}}_{k}+a_{i}=\overset{i}{\underset{k=1}{\sum }}a_{k}⩽A_{\mathrm{GG}}.\;□$$

### 3.2 Performance of the Look-ahead Greedy Algorithm

We start with a simple proposition complementing Proposition 2.

Proposition 8 *It can always be assumed that the L*ook-ahead *G*reedy *strategy of agent P*_*a*_ *selects items in nonincreasing order of weights against the optimal strategy of agent P*_*b*_*.*

Proof Assume that for some round *j* there is *a*_*j*_ < *a*_*j*+1_. Since in round *j* the Look-ahead Greedy computes a pair of items and selects the larger of the pair, this means that a different pair (*j*, *k*) was determined yielding *p*_max_. By definition of the algorithm *a*_*j*_ ⩾ *a*_*k*_ holds and hence *a*_*k*_ < *a*_*j*+1_. Hence, items *j* and *j* + 1 would have been a better pair than *j* and *k* and must have been excluded from the computation of *p*_max_, which means that $a_{j}+b_{\max}+a_{j+1}>\overset{¯}{c}$.

By definition of *b*_max_ in Look-ahead Greedy, the item with weight *b*_max_ would be a feasible selection for *P*_*b*_ after *a*_*j*_. But then

(6) $$b_{\max}>c-a_{j}-a_{j+1}⩾c-A_{\mathrm{LO}}⩾B_{\mathrm{LO}}$$

yields a contradiction to the optimality of the strategy of *P*_*b*_. □

Note that Proposition 8 does not hold for every adversary strategy *S*. *P*_*b*_ may surprisingly select an extremely small item thus permitting *P*_*a*_ to select a larger item in round two and deviate from the original strategy to select a certain pair of items, each one with smaller weight.

Theorem 9 *The L*ook-ahead *G*reedy *algorithm L has a tight performance bound of*

$$\rho_{\mathrm{LO}}=\frac{2}{3}\text{.}$$

Proof Let *Opt*≔*A*_*OO*_ and assume *A*_*OO*_ > *A*_*LO*_ (otherwise *A*_*OO*_ = *A*_*LO*_ and we are done). Denote the items selected by an optimal strategy *O* of *P*_*a*_ as ${\overset{¯}{a}}_{j}$. By Proposition 8, *a*_1_ and *a*_2_ are the largest two items selected by *L*. If $a_{1}+a_{2}⩾\frac{2}{3}\;\mathrm{Opt}$ we are done. Assuming $a_{1}+a_{2}<\frac{2}{3}\;\mathrm{Opt}$ it follows again from Proposition 8 that $a_{j}<\frac{1}{3}\;\mathrm{Opt}$ for all *j* ⩾ 2.

First, we consider the special case ${\overset{¯}{a}}_{1}>a_{1}$: It follows from the decision of *L* in the first round that ${\overset{¯}{a}}_{1}$ and *a*_2_ cannot be considered in the selection of the best pair, because this pair would be better than *a*_1_ and *a*_2_, but ${\overset{¯}{a}}_{1}$, as the larger of the pair, was not selected in round 1. Thus, there must exist some item $\overset{˜}{b}$, which *P*_*b*_ could select in round 1, to block this pair, i.e. ${\overset{¯}{a}}_{1}+\overset{˜}{b}⩽c$ and ${\overset{¯}{a}}_{1}+a_{2}+\overset{˜}{b}>c$. Clearly, this means that $A_{\mathrm{OO}}⩽c-\overset{˜}{b}$. Hence, we have

$$A_{\mathrm{OO}}-A_{\mathrm{LO}}⩽c-\overset{˜}{b}-(a_{1}+a_{2})<{\overset{¯}{a}}_{1}-a_{1}\text{.}$$

Now we note that ${\overset{¯}{a}}_{1}<\frac{2}{3}\;\mathrm{Opt}$ because otherwise ${\overset{¯}{a}}_{1}$ would be a sufficiently large solution for *L* as a single item.

If $a_{1}⩾\frac{1}{3}\;\mathrm{Opt}$, then we have from above

$$A_{\mathrm{OO}}-A_{\mathrm{LO}}<{\overset{¯}{a}}_{1}-a_{1}<\frac{2}{3}\;\mathrm{Opt}-\frac{1}{3}\;\mathrm{Opt}=\frac{1}{3}\;\mathrm{Opt}$$

and are done.

If $a_{1}<\frac{1}{3}\;\mathrm{Opt}$, we can use the previous inequalities to show

$${\overset{¯}{a}}_{1}+a_{1}⩾{\overset{¯}{a}}_{1}+a_{2}>c-\overset{˜}{b}⩾A_{\mathrm{OO}}=\mathrm{Opt}\text{.}$$

Since ${\overset{¯}{a}}_{1}<\frac{2}{3}\;\mathrm{Opt}$ this implies $a_{1}>\frac{1}{3}\;\mathrm{Opt}$ in contradiction to the assumption of this subcase.

Generalizations of these arguments will be used in the following to show the statement for the general case of ${\overset{¯}{a}}_{1}⩽a_{1}$.

At first we introduce two technical lemmata. Lemma 10 corresponds to the situation of *P*_*a*_ trying to select a pair $\overset{˜}{a}$ and $a_{j^{\prime }+1}$ in round *j*′ + 1 and realizing that $\overset{˜}{a}$ could well be packed on its own, but *P*_*b*_ has an item $\overset{˜}{b}$ available to block $a_{j^{\prime }+1}$. □

Lemma 10 *Assume that after completion of some round j*′*, j*′ < *n*_*a*_ *of P*_*a*_ *playing L against O of P*_*b*_*, there exist items* $\overset{˜}{a}$ *(resp.* $\overset{˜}{b}$*) not yet selected by L (resp. P*_*b*_*), such that the following inequalities hold:*

(7) $$\overset{j^{\prime }}{\underset{k=1}{\sum }}a_{k}+\overset{˜}{a}+\overset{j^{\prime }}{\underset{k=1}{\sum }}b_{k}+\overset{˜}{b}⩽c\text{,}$$

(8) $$\overset{j^{\prime }}{\underset{k=1}{\sum }}a_{k}+\overset{˜}{a}+a_{j^{\prime }+1}+\overset{j^{\prime }}{\underset{k=1}{\sum }}b_{k}+\overset{˜}{b}>c\text{.}$$

*If* $B_{\mathrm{OO}}⩾\sum_{k=1}^{j^{\prime }}b_{k}+\overset{˜}{b}$ *then we have*

$$A_{\mathrm{OO}}-A_{\mathrm{LO}}<\overset{˜}{a}\text{.}$$

Proof Clearly, we can bound the weight obtained by *L* as $A_{\mathrm{LO}}⩾\sum_{k=1}^{j^{\prime }+1}a_{k}$. Then we get from the condition of the Lemma and by applying (8)

$$A_{\mathrm{OO}}-A_{\mathrm{LO}}⩽(c-B_{\mathrm{OO}})-\overset{j^{\prime }+1}{\underset{k=1}{\sum }}a_{k}<c-\overset{j^{\prime }}{\underset{k=1}{\sum }}b_{k}-\overset{˜}{b}-\left(c-\overset{j^{\prime }}{\underset{k=1}{\sum }}b_{k}-\overset{˜}{b}-\overset{˜}{a}\right)=\overset{˜}{a}.\;□$$

Lemma 11 states that as long as *L* dominates the optimal strategy of *P*_*a*_ in every round, the total weight collected by *P*_*b*_ against an optimal strategy of *P*_*a*_ is at least as large as the weight *P*_*b*_ would gain against an adversary *P*_*a*_ pursuing strategy *L*.

Lemma 11 *If there exists an index j*′ ⩾ *1 such that*

(9) $$\overset{\ell }{\underset{k=1}{\sum }}a_{k}⩾\overset{\ell }{\underset{k=1}{\sum }}{\overset{¯}{a}}_{k}\;\mathrm{for}\;\mathrm{all}\;\ell =1\text{,}\ldots \text{,}j^{\prime }\text{,}$$

*then* $B_{\mathrm{OO}}⩾\sum_{k=1}^{j^{\prime }}b_{k}$*.*

Proof If $B_{\mathrm{OO}}<\sum_{k=1}^{j^{\prime }}b_{k}$, then the strategy of *P*_*b*_ cannot be optimal since *P*_*b*_ could have chosen the items $b_{1}\text{,}\ldots \text{,}b_{j^{\prime }}$ instead. By the condition of the lemma these items would have been a feasible choice in every round ℓ ⩽ *j*′. □

Proceeding with the proof of Theorem 9 we now let *j* be the first round where the optimal strategy reaches a higher total weight than *L*, i.e. *j* is the minimal index such that

(10) $$\overset{j-1}{\underset{k=1}{\sum }}a_{k}⩾\overset{j-1}{\underset{k=1}{\sum }}{\overset{¯}{a}}_{k}\;\mathrm{and}\;\overset{j}{\underset{k=1}{\sum }}a_{k}<\overset{j}{\underset{k=1}{\sum }}{\overset{¯}{a}}_{k}\text{.}$$

Note that *j* ⩾ 2 holds in the considered case of ${\overset{¯}{a}}_{1}⩽a_{1}$.

Now we consider the item set *D* consisting of items selected in the first *j* rounds by *O*, but not by *L*, i.e. $D≔\{{\overset{¯}{a}}_{1}\text{,}\ldots \text{,}{\overset{¯}{a}}_{j}\}⧹\{a_{1}\text{,}\ldots \text{,}a_{j}\}$. Obviously, *D* ≠ ∅. Let ${\overset{¯}{a}}_{D}^{1}$ (resp. ${\overset{¯}{a}}_{D}^{2}$) denote the largest (resp. second largest, if it exists) item in *D*. Note that ${\overset{¯}{a}}_{D}^{2}$ will only be used in the proof if ∣*D*∣ ⩾ 2.

**Case 1**: ${\overset{¯}{a}}_{D}^{1}⩽\frac{1}{3}\mathrm{Opt}$. Trivially, ${\overset{¯}{a}}_{D}^{1}>a_{j}$ because of (10). Recall from Proposition 8 that *a*_*j*_ is the smallest item selected by *L* in the first *j* rounds. Considering the decision of *L* in round *j* − 1 we distinguish two cases:
  **Case 1.1**: ${\overset{¯}{a}}_{D}^{1}>a_{j-1}$. In this case, *L* was able to select a pair consisting of *a*_*j*−1_ and some other item with smaller weight, possibly *a*_*j*_ but maybe some other item, in round *j* − 1. However, the better pair ${\overset{¯}{a}}_{D}^{1}$ and *a*_*j*−1_ was not selected because otherwise ${\overset{¯}{a}}_{D}^{1}$ would have been selected by *L* in round *j* − 1 as the larger item of the pair. This omission of ${\overset{¯}{a}}_{D}^{1}$ by *L* must be caused by one of the following two cases:
    (a) ${\overset{¯}{a}}_{D}^{1}$ could not be added in round *j* − 1 at all. But this means that
      (11) $$\overset{j-2}{\underset{k=1}{\sum }}a_{k}+{\overset{¯}{a}}_{D}^{1}+\overset{j-2}{\underset{k=1}{\sum }}b_{k}>c\text{.}$$
      Since *j* was defined as the smallest index fulfilling (10), we can apply Lemma 11 for *j*′ = *j* − 2 (also *j*′ = *j* − 1 would be feasible, but is not required here) and get $B_{\mathrm{OO}}⩾\sum_{k=1}^{j-2}b_{k}$. Replicating the proof of Lemma 10 and plugging in (11) it follows that
      (12) $$A_{\mathrm{OO}}-A_{\mathrm{LO}}⩽c-B_{\mathrm{OO}}-\overset{j-2}{\underset{k=1}{\sum }}a_{k}<c-\overset{j-2}{\underset{k=1}{\sum }}b_{k}-\left(c-\overset{j-2}{\underset{k=1}{\sum }}b_{k}-{\overset{¯}{a}}_{D}^{1}\right)={\overset{¯}{a}}_{D}^{1}⩽\frac{1}{3}\;\mathrm{Opt}$$
      and we are done.
    (b) ${\overset{¯}{a}}_{D}^{1}$ could be added in round *j* − 1, but (according to the definition of *L*) *P*_*b*_ has some “blocking” item $\overset{˜}{b}$ available preventing *P*_*a*_ from selecting ${\overset{¯}{a}}_{D}^{1}$ in round *j* − 1 and *a*_*j*−1_ in round *j*. Choosing *j*′ = *j* − 2 and $\overset{˜}{a}={\overset{¯}{a}}_{D}^{1}$ this blocking scenario is exactly described by (7) and (8).
      Condition (9) of Lemma 11 clearly holds for ℓ = 1, … , *j* − 2, but can be extended for ℓ = *j* − 1 as follows: With (10) we can state that
      $$\overset{j-2}{\underset{k=1}{\sum }}a_{k}+{\overset{¯}{a}}_{D}^{1}>\overset{j-1}{\underset{k=1}{\sum }}a_{k}⩾\overset{j-1}{\underset{k=1}{\sum }}{\overset{¯}{a}}_{k}\text{.}$$
      For our case, (7) says that even if in round *j* − 1 agent *P*_*a*_ selected an item ${\overset{¯}{a}}_{D}^{1}$ larger than *a*_*j*−1_, agent *P*_*b*_ could still add $\overset{˜}{b}$ to its current selection of $\sum_{k=1}^{j-2}b_{k}$. Replicating the proof of Lemma 11 this yields $B_{\mathrm{OO}}⩾\sum_{k=1}^{j-2}b_{k}+\overset{˜}{b}$. Therefore, the condition of Lemma 10 is fulfilled and we are done since $A_{\mathrm{OO}}-A_{\mathrm{LO}}<{\overset{¯}{a}}_{D}^{1}⩽\frac{1}{3}\mathrm{Opt}$.
  **Case 1.2**: ${\overset{¯}{a}}_{D}^{1}⩽a_{j-1}$. In this case we notice that among the items selected by *L*, but not by *O*, *a*_*j*_ is the only item smaller than ${\overset{¯}{a}}_{D}^{1}$ (the largest item in *D*). Hence, the difference between the weights of *L* and *O* after round *j* can be at most ${\overset{¯}{a}}_{D}^{1}-a_{j}$, since all other items in *D* only diminish this difference. Formally,
    (13) $$\overset{j}{\underset{k=1}{\sum }}{\overset{¯}{a}}_{k}-\overset{j}{\underset{k=1}{\sum }}a_{k}⩽{\overset{¯}{a}}_{D}^{1}-a_{j}\text{.}$$
    As in Case 1.1, there are two possible scenarios why *L* did not choose ${\overset{¯}{a}}_{D}^{1}$ in round *j* but settled for the smaller item *a*_*j*_:
    (a) If ${\overset{¯}{a}}_{D}^{1}$ could not be added in round *j* at all, then we can repeat the arguments of Case 1.1 (a). Exchanging *j* − 2 by *j* − 1 we apply (11) and (12) verbatim and are done.
    (b) If ${\overset{¯}{a}}_{D}^{1}$ could be added in round *j*, we recall Case 1.1 (b) and note that *P*_*b*_ has again some “blocking” item $\overset{˜}{b}$ available to prevent the pair ${\overset{¯}{a}}_{D}^{1}$ and *a*_*j*_, i.e. fulfilling (7) and (8) for *j*′ = *j* − 1 and $\overset{˜}{a}={\overset{¯}{a}}_{D}^{1}$.
      Now, (10) guarantees the condition required in Lemma 11 only for ℓ = 1, … , *j* − 1. With (13) it can be extended for ℓ = *j* since $\sum_{k=1}^{j}{\overset{¯}{a}}_{k}⩽\sum_{k=1}^{j-1}a_{k}+{\overset{¯}{a}}_{D}^{1}$. Now (7) says that even if in round *j* agent *P*_*a*_ selected an item ${\overset{¯}{a}}_{D}^{1}$ larger than *a*_*j*_, agent *P*_*b*_ could still add $\overset{˜}{b}$ to its current selection of $\sum_{k=1}^{j-1}b_{k}$. Again replicating the proof of Lemma 11 yields $B_{\mathrm{OO}}⩾\sum_{k=1}^{j-1}b_{k}+\overset{˜}{b}$. Now the condition of Lemma 10 is fulfilled and $A_{\mathrm{OO}}-A_{\mathrm{LO}}<{\overset{¯}{a}}_{D}^{1}⩽\frac{1}{3}\mathrm{Opt}$ settles this case.
**Case 2:** ${\overset{¯}{a}}_{D}^{1}>\frac{1}{3}\mathrm{Opt}$. If $a_{2}⩾\frac{1}{3}\mathrm{Opt}$ then $a_{1}+a_{2}⩾\frac{2}{3}\mathrm{Opt}$ and we are done. Hence, in the following we can assume $a_{2}<\frac{1}{3}\mathrm{Opt}<{\overset{¯}{a}}_{D}^{1}$. Furthermore, we first consider the special case that *a*_1_ was also selected by *O*, i.e. *O* contains both *a*_1_ and ${\overset{¯}{a}}_{D}^{1}$. In that case, this pair is also a feasible pair for *L* to consider in the first round. If *P*_*b*_ were able to block this pair, it could increase its weight by doing so and thus *O* could not select both against an optimal strategy of *P*_*b*_. If $a_{1}⩾{\overset{¯}{a}}_{D}^{1}$, then $a_{1}+{\overset{¯}{a}}_{D}^{1}⩾\frac{2}{3}\mathrm{Opt}$ and we are done. If $a_{1}<{\overset{¯}{a}}_{D}^{1}$ then ${\overset{¯}{a}}_{D}^{1}$ and *a*_1_ are a better pair than the pair selected by *L* in the first round consisting of *a*_1_ and some smaller item *a*′. *L* selected *a*_1_ as the larger of the smaller pair (*a*_1_, *a*′), but by definition *L* should have selected ${\overset{¯}{a}}_{D}^{1}$ as the larger item of the better pair $\left({\overset{¯}{a}}_{D}^{1}\text{,}a_{1}\right)$ in the first round. Hence, we can assume in the following that *a*_1_ was not selected by *O*.
  **Case 2.1**: ∣*D*∣ = 1. In order to satisfy (10) we must have $a_{1}<{\overset{¯}{a}}_{D}^{1}$ in this case, since items *a*_2_, … , *a*_*j*_ are selected both by *L* and by *O*. Therefore, ${\overset{¯}{a}}_{D}^{1}$ and *a*_2_ are both contained in *O* and they are a better feasible pair than *a*_1_ and *a*_2_. Since *P*_*b*_ could not prevent this pair (otherwise it would be have been advantageous for *P*_*b*_ to do so), *L* should have selected ${\overset{¯}{a}}_{D}^{1}$ in the first round as the larger item of the feasible pair $\left({\overset{¯}{a}}_{D}^{1}\text{,}a_{2}\right)$ instead of *a*_1_. Thus, we have found a contradiction to the definition of *L*.
  **Case 2.2**: ∣*D*∣ ⩾ 2. We can assume ${\overset{¯}{a}}_{D}^{2}<\frac{1}{3}\mathrm{Opt}$, because otherwise *L* could have selected ${\overset{¯}{a}}_{D}^{1}$ and ${\overset{¯}{a}}_{D}^{2}$ in the first round and reach at least $\frac{2}{3}\mathrm{Opt}$. Depending on the size of *a*_1_ we distinguish two subcases:
    (a) $a_{1}<{\overset{¯}{a}}_{D}^{1}$. Then ${\overset{¯}{a}}_{D}^{1}$ and *a*_2_ would be a better pair than *a*_1_ and *a*_2_, but they are not selected by *L* in the first round. This is due to the fact that *P*_*b*_ is able to block this pair with some item $\overset{˜}{b}$ with ${\overset{¯}{a}}_{D}^{1}+a_{2}+\overset{˜}{b}>c$ and ${\overset{¯}{a}}_{D}^{1}+\overset{˜}{b}⩽c$. The latter means that $\overset{˜}{b}$ fits also against the largest item of *O*. Hence, combining the two conditions yields $A_{\mathrm{OO}}⩽c-\overset{˜}{b}<{\overset{¯}{a}}_{D}^{1}+a_{2}$. Since ${\overset{¯}{a}}_{D}^{1}<\frac{2}{3}\mathrm{Opt}$, this implies $a_{2}>\frac{1}{3}\mathrm{Opt}$ and thus $a_{1}+a_{2}>\frac{2}{3}\mathrm{Opt}$ and we are done.
    (b) $a_{1}⩾{\overset{¯}{a}}_{D}^{1}$. Let *a*^∗^ be the largest item from *a*_2_, … , *a*_*j*_ not contained in *O* with $a^{*}<{\overset{¯}{a}}_{D}^{2}$, i.e. $a^{*}=\max\left\{a_{k}|a_{k}<{\overset{¯}{a}}_{D}^{2}\text{,}k=2\text{,}\ldots \text{,}j\right\}$. Such an *a*^∗^ must exist, because otherwise all items *a*_2_, … , *a*_*j*_ would be greater or equal ${\overset{¯}{a}}_{D}^{2}$ and $a_{1}⩾{\overset{¯}{a}}_{D}^{1}$ which makes fulfillment of (10) impossible.
      If *a*^∗^ was selected by *L* in some round *j*′ < *j*, we can simply repeat without changes the arguments of Case 1.1 for *j*′ instead of *j* − 1 and with ${\overset{¯}{a}}_{D}^{2}$ replacing ${\overset{¯}{a}}_{D}^{1}$. Recalling that ${\overset{¯}{a}}_{D}^{2}<\frac{1}{3}\mathrm{Opt}$ we reach the desired result.
      If *a*^∗^ was selected by *L* in round *j*, i.e. *a*^∗^ = *a*_*j*_, we have (recall Proposition 8) $a_{2}⩾\ldots ⩾a_{j-1}⩾{\overset{¯}{a}}_{D}^{2}>a^{*}$. Since also $a_{1}⩾{\overset{¯}{a}}_{D}^{1}$, the difference between the weights of *L* and *O* up to round *j* can be at most ${\overset{¯}{a}}_{D}^{2}-a^{*}$, in analogy to (13). Now we study the treatment of ${\overset{¯}{a}}_{D}^{2}$.
      (b1) ${\overset{¯}{a}}_{D}^{2}$ could not be added by *L* in round *j* (cf. Case 1.1(a)). This means that
        $$\overset{j-1}{\underset{k=1}{\sum }}a_{k}+{\overset{¯}{a}}_{D}^{2}+\overset{j-1}{\underset{k=1}{\sum }}b_{k}>c\text{.}$$
        Because of (10), Lemma 11 can be applied with *j*′ = *j* − 1 yielding $B_{\mathrm{OO}}⩾\sum_{k=1}^{j-1}b_{k}$. Replicating again the proof of Lemma 10 in analogy to (12) we get with the above
        (14) $$A_{\mathrm{OO}}-A_{\mathrm{LO}}⩽c-B_{\mathrm{OO}}-\overset{j-1}{\underset{k=1}{\sum }}a_{k}<c-\overset{j-1}{\underset{k=1}{\sum }}b_{k}-\left(c-\overset{j-1}{\underset{k=1}{\sum }}b_{k}-{\overset{¯}{a}}_{D}^{2}\right)={\overset{¯}{a}}_{D}^{2}⩽\frac{1}{3}\;\mathrm{Opt}\text{.}$$
      (b2) ${\overset{¯}{a}}_{D}^{2}$ could be added by *L* in round *j* (cf. Case 1.1(b)). Since *L* selected *a*_*j*_ in round *j* together with some other, smaller item, we know that the better pair $\left({\overset{¯}{a}}_{D}^{2}\text{,}a_{j}\right)$ was not chosen in round *j*, although ${\overset{¯}{a}}_{D}^{2}$ would fit on its own. Thus, there must be again some blocking item $\overset{˜}{b}$ available for *P*_*b*_ fulfilling (7) and (8) for *j*′ = *j* − 1 with $\overset{˜}{a}={\overset{¯}{a}}_{D}^{2}$.
      As before, condition (9) of Lemma 11 holds for ℓ = 1, … , *j* − 1 and can be extended for ℓ = *j*: Recall from above the bound on the difference between the weight of *L* and *O* with *a*^∗^ = *a*_*j*_,
      $$\overset{j}{\underset{k=1}{\sum }}{\overset{¯}{a}}_{k}-\overset{j}{\underset{k=1}{\sum }}a_{k}⩽{\overset{¯}{a}}_{D}^{2}-a_{j}\Rightarrow \overset{j-1}{\underset{k=1}{\sum }}a_{k}+{\overset{¯}{a}}_{D}^{2}⩾\overset{j}{\underset{k=1}{\sum }}{\overset{¯}{a}}_{k}\text{.}$$
      Replicating again the proof of Lemma 11 and considering (7) it follows that $B_{\mathrm{OO}}⩾\sum_{k=1}^{j-1}b_{k}+\overset{˜}{b}$. Therefore, Lemma 10 can be applied and we are done since
      $$A_{\mathrm{OO}}-A_{\mathrm{LO}}<{\overset{¯}{a}}_{D}^{2}⩽\frac{1}{3}\mathrm{Opt}\text{.}$$

All together we have shown $\rho_{\mathrm{LO}}⩽\frac{2}{3}$.

The following Example 5 is a straightforward extension of Example 3. It gives an instance with parameter *ε* > 0 where $\lim_{\varepsilon \to 0}\rho_{\mathrm{LO}}=\frac{2}{3}$ thus completing the proof of the theorem.

Example 5 Consider an instance of our problem in which the capacity is *c* = 1 and the items weights are as follows.

**Table:** 

Item	1	2	3	4	5
*N*_*a*_	$\frac{1}{3}$	$\frac{1}{3}$	$\frac{1}{3}-\varepsilon$	$\frac{1}{3}-\varepsilon$	$\frac{1}{3}-\varepsilon$
*N*_*b*_	2*ε*	*ε*	*ε*		

In the first two rounds *P*_*a*_ by the Look-ahead Greedy strategy selects items 1 and 2 with total weight $A_{\mathrm{LO}}=\frac{2}{3}$ while *P*_*b*_ gains 3*ε*. After the second round *P*_*a*_ cannot select another item.

An optimal strategy would select items 3, 4 and 5 with a total weight of *A*_*OO*_ = 1 − 3*ε*. □

### 3.3 Performance of the *k*-Look-ahead Greedy Algorithm

It is natural to expect that the performance of the Look-ahead Greedy heuristic should improve the more items one includes in the look ahead set, i.e. *ρ*_*k*−LO_ increases in *k*. When moving from *k* = 1 to *k* = 2 this was shown to be true in the previous sections. The general case could be seen as being related to the construction of a polynomial time approximation scheme (PTAS) for the Subset Sum and the Knapsack problem, where subsets of a certain cardinality ℓ are enumerated and the larger ℓ, the smaller the resulting relative error *ε*.

Surprisingly, the following example shows that this is not the case. Instead, it can be shown that the worst case performance bound of $\frac{2}{3}$ given in Theorem 9 is also an upper bound for the *k*-Look-ahead Greedy algorithm for arbitrary *k* ⩾ 3.

Theorem 12 *The performance of the k-L*ook-ahead *G*reedy *algorithm k* − *L for k* ⩾ *3 is bounded by*

$$\rho_{k-\mathrm{LO}}⩽\frac{2}{3}\text{.}$$

Proof The theorem can be proven by considering an instance with capacity *c* = 12, *δ* ≪ *ε*, *k* ∈ {3, … , *n*_*a*_} and the following items weights.

**Table:** 

Item	1	2	3	4	5	6	⋯	*n*_*a*_

*N*_*a*_	2 + *ε*	2 + *ε*	2 − *ε*	2 − *ε*	2 − *ε*	*δ*	⋯	*δ*
*N*_*b*_	6	3 + *ε*	3 + *ε*	$2+\frac{1}{2}\varepsilon$

*P*_*a*_ is able to choose any pair of items from the set {1, 2, 3, 4, 5} together with items of size *δ* in the first round, since they cannot be blocked by *P*_*b*_. However *P*_*a*_ is neither able to choose any set containing items 1, 2, 3 (which would exceed the capacity) nor can *P*_*a*_ choose items 3, 4 and 5, as *P*_*b*_ can block them with items 1 and 4. Hence the set of items considered by *P*_*a*_ in the first round of any *k*-Look-ahead Greedy contains one item of weight 2 + *ε* and two items of weight 2 − *ε*. Thus, item 1 is selected in the first round. A residual capacity $\overset{¯}{c}=10-\varepsilon$ remains for *P*_*b*_ who has three possibilities to react. The resulting games are listed as columns in the following table:

**Table:** 

Round 1 of *P*_*a*_	2 + *ε*	2 + *ε*	2 + *ε*
Round 1 of *P*_*b*_	6	3 + *ε*	$2+\frac{1}{2}\varepsilon$

$\overset{¯}{c}$	4 − *ε*	7 − 2*ε*	$8-\frac{3}{2}\varepsilon$
Best tuple of *P*_*a*_	2 − *ε*, 2 − *ε*	2 + *ε*, *δ*	2 + *ε*, 2 − *ε*
Round 2 of *P*_*a*_	2 − *ε*	2 + *ε*	2 + *ε*
$\overset{¯}{c}$	2	5 − 3*ε*	$6-\frac{5}{2}\varepsilon$
Round 2 of *P*_*b*_		3 + *ε*	3 + *ε*

$\overset{¯}{c}$	*2*	2 − 4*ε*	$3-\frac{7}{2}\varepsilon$
Round 3 of *P*_*a*_	2 − *ε*	*δ*	2 − *ε*
Round 3 of *P*_*b*_			

*A*_*k*−LO_	6 − *ε* + (*n*_*a*_ − 3)*δ*	4 + 2*ε* + (*n*_*a*_ − 2)*δ*	6 + *ε* + (*n*_*a*_ − 3)*δ*
*B*_*k*−LO_	6	6 + 2*ε*	$5+\frac{3}{2}\varepsilon$

It turns out that the optimal strategy for *P*_*b*_ against the *k*-Look-ahead Greedy of *P*_*a*_ is given by selecting item 2 in the first round as represented in the second column of the table. Thus, we get *A*_*k*−LO_ ≈ 4

The following table shows the optimal strategy of *P*_*a*_ against an optimal strategy of *P*_*b*_. *P*_*a*_ starts the game by selecting an item of weight 2 − *ε* leaving a residual capacity $\overset{¯}{c}=10+\varepsilon$ for *P*_*b*_. For *P*_*b*_ three possibilities remain:

**Table:** 

Round 1 of *P*_*a*_	2 − *ε*	2 − *ε*	2 − *ε*
Round 1 of *P*_*b*_	6	3 + *ε*	$2+\frac{1}{2}\varepsilon$

$\overset{¯}{c}$	4 + *ε*	7	$8+\frac{1}{2}\varepsilon$
Round 2 of *P*_*a*_	2 + *ε*	2 − *ε*	2 + *ε*
$\overset{¯}{c}$	2	5 + *ε*	$6-\frac{1}{2}\varepsilon$
Round 2 of *P*_*b*_		3 + *ε*	3 + *ε*

$\overset{¯}{c}$	2	2	$3-\frac{3}{2}\varepsilon$
Round 3 of *P*_*a*_	2 − *ε*	2 − *ε*	2 + *ε*
Round 3 of *P*_*b*_			

*A*_*OO*_	6 − *ε* + (*n*_*a*_ − 3)*δ*	6 − 3*ε* + (*n*_*a*_ − 3)*δ*	6 + *ε* + (*n*_*a*_ − 3)*δ*
*B*_*OO*_	6	6 + 2*ε*	$5+\frac{3}{2}\varepsilon$

Again, the optimal strategy for *P*_*b*_ is given by selecting item 2 in the first round as illustrated in the second column. We get *A*_*OO*_ ≈ 6 which completes the proof. □

## 4 The centralized perspective

As it is often done in game theoretic settings, we can put the outcome of the game by two competing and selfish agents in perspective to a centralized view, where a single decision maker makes all selections for both item sets *N*_*a*_ and *N*_*b*_. The goal of such a centralized decision is the maximization of the total weight obtained from both item sets. Clearly, the centralized decision has to select items from *N*_*a*_ and *N*_*b*_ in turn as in the underlying Subset Sum game.

It is easy to see that the computation of such a globally optimal solution weight *W*^∗^ is an NP-hard problem since it contains the classical Subset Sum problem (SSP). A reduction can be obtained similar to the proof of Proposition 1.

In algorithmic game theory, the Prize of Anarchy is a widely used concept to analyze the difference between a global optimum and the outcome arising from the combined solutions of selfish agents. For the Subset Sum game, we compare the optimal weight generated by the central decision to the outcome obtained by the two agents each following its own optimal strategy (cf. Section 2.1). A simple example shows that the Prize of Anarchy can be arbitrarily high.

Proposition 13 *There exist instances where*

$$\frac{W^{*}}{A_{\mathrm{OO}}+B_{\mathrm{OO}}}\to \infty \text{.}$$

Example 6 Consider an instance with capacity *c* = 1 and the following sets of items.

**Table:** 

Item	1	2
*N*_*a*_	2*ε*	*ε*
*N*_*b*_	1 − *ε*	*ε*

A centralized optimal solution would select item 2 from *N*_*a*_ and item 1 from *N*_*b*_ in the first round and reach *W*^∗^ = 1. An optimal strategy by *P*_*a*_ would surely start with item 1 which leaves to *P*_*b*_ only the selection of item 2 and *A*_*OO*_ + *B*_*OO*_ = 3*ε*.

The central decision maker could also consider the game as a bicriteria optimization problem where the items from each agent’s set constitute one objective. The decision problem whether a certain pair of weights (*W*_*a*_, *W*_*b*_) can be reached is again NP-complete from SSP.

From an approximation point of view, it is not sufficient to apply a fully polynomial approximation scheme (FPTAS) for each (SSP) associated to each agent since one has to take the rounds with alternating selections from both item sets into account. However, we can consider the *cardinality constrained Subset Sum problem* (kSSP), where at most *k* items can be selected. Following the dynamic programming approach in Caprara, Kellerer, Pferschy, and Pisinger (2000) and assuming integer weights, we can compute for each agent every reachable pair (ℓ, *W*) with ℓ = 1, … , *n*_*a*_, respectively *n*_*b*_, and *W* = 1, … , *c*. Then we search for the best combination of two solutions with equal cardinality (=number of rounds) such that agent’s *P*_*a*_ weights reach at least *W*_*a*_ and agent’s *P*_*b*_ weights reach at least *W*_*b*_ but their sum does not exceed *c*.

Now we can transform this pseudopolynomial exact dynamic programming solution procedure into an FPTAS by the usual scaling techniques (cf. Caprara et al., 2000) and thus answer the approximation version of the above question whether a solution with weights (*A*, *B*) exists with (1 − *ε*)*W*_*a*_ ⩽ *A* ⩽ *W*_*a*_ and (1 − *ε*)*W*_*b*_ ⩽ *B* ⩽ *W*_*b*_ in time polynomial in the size of the encoded input and 1/*ε*.

## 5 Extension to the Knapsack Game

It appears natural to extend the addressed Subset Sum game to a Knapsack Game by introducing profits for all items. In this case, the two agents would strive to maximize the total profit of their selected items while the weights still have to obey the capacity restriction. In this section it is shown that extending the Greedy-type algorithms introduced in Section 2.2 to the knapsack case does not yield a bounded performance ratio.

There are two natural approaches to extend the Greedy or *k*-Look-ahead Greedy algorithms to a Knapsack Game: one may, in each step, choose the most “efficient” items (according to their profit to weight ratio), or one tries to gain as much profit as possible by simply choosing the item with largest profit in each round.

It can easily be shown that the *k*-Look-ahead Greedy (*k* ⩾ 2) – which stepwise lays its focus on the best *k*-tuple according to the *sum of the profit to weight ratios* – has an arbitrarily bad performance bound. The same example works also for *k* = 1 which corresponds to the Greedy algorithm.

Example 7 Consider the following instance of the problem with *c* = 1.

**Table:** 

Item		1	⋯	*k*	*k* + 1
*N*_*a*_	profit	2*ε*	⋯	2*ε*	1
	weight	*ε*	⋯	*ε*	1 − *ε*
*N*_*b*_	profit	1			
	weight	1 − *kε*			

Sorting by profit to weight ratios, *P*_*a*_ would identify the items 1, … , *k* as the best *k*-tuple and select one of them in the first round. This leaves *P*_*b*_ the chance to submit its only item. The residual capacity of (*k* − 1)*ε* can be used by *P*_*a*_ to select all remaining items 2, … , *k*. Hence, the game stops with a total profit of 2*kε* for *P*_*a*_ against any strategy of *P*_*b*_. An optimal strategy of *P*_*a*_ would select item *k* + 1 in first round and item 1 in the second round gaining a profit of 1 + 2*ε* while *P*_*b*_ cannot select any item.

The following example shows that also the *k*-Look-ahead Greedy algorithm for *k* ⩾ 1, i.e. including the pure Greedy algorithm, has an arbitrarily bad performance bound when focusing on the *k*-tuple with the *highest total profit* in each round.

Example 8 Consider the following instance with *n* ≫ *k* and *c* = 1 + *ε*.

**Table:** 

Item		1	⋯	*k*	*k* + 1	⋯	*n*
*N*_*a*_	profit	$k+\frac{k+1}{n-k}$	⋯	$k+\frac{k+1}{n-k}$	$1+\frac{1}{n-k}$	⋯	$1+\frac{1}{n-k}$
	weight	$\frac{1}{k}$	⋯	$\frac{1}{k}$	$\frac{1}{n-k}$	⋯	$\frac{1}{n-k}$
*N*_*b*_	profit	1					
	weight	*ε*					

Sorting by profits, similarly to Example 7, *P*_*a*_ may select item 1 in the first round while *P*_*b*_ selects its only item. In all *k* − 1 remaining rounds, *P*_*a*_ chooses a *k*-tuple with all remaining items from 2, … , *k* complemented by smaller items and thus selects a large item in each round yielding a total profit of $k^{2}+\frac{k(k+1)}{n-k}$ for any strategy *S* of *P*_*b*_. An optimal strategy of *P*_*a*_ would select items *k* + 1, … , *n* and gain a profit of (*n* − *k*) + 1. For *n* tending to infinity, the performance of the profit based on *k*-Look-ahead Greedy becomes arbitrarily bad.

## 6 Conclusions

In this paper we have analyzed a game theoretic variant of the well known Subset Sum problem in which the agents take turns to select their items to be included in a capacitated shared resource. This round-robin dynamics has been considered in other game theoretic studies of classical combinatorial problems (Agnetis et al., 2007; Auletta et al., 2009; Piliouras, Valla, & Vegh, 2012), however a possible topic for further research is to consider, as in Marini et al. (2013) and Nicosia et al. (2011), a different game theoretic variant of the Subset Sum problem where the round-robin mechanism is replaced by a central decision mechanism (coordinator) which picks only one of the two items selected by the two agents in each round (cf. Section 1.2). Hence, a coordinator may decide to enforce different rules, that incorporate some idea of fairness (which is for instance very important in the context of telecommunication networks).

Another possible direction for future research is to analyze the existence and the properties of equilibria, which can be used by a coordinator to enforce solutions from which the agents have no incentive to deviate.

Finally, one could study the problem in which an agent has limited information on the other agent’s items.

## Figures and Tables

**Table 1:** Resulting games in instance I, after *P*_*a*_ chose item 1 in the first round.

Round 1 of *P*_*b*_	5 + 4*ε*	5	2	3*ε*
$\overset{¯}{c}$	8 − *ε*	8 + 3*ε*	11 + 3*ε*	13
Best pair of *P*_*a*_	6, 0	3 + *ε*, 3 + *ε*	6, 0	3 + *ε*, 3 + *ε*
Round 2 of *P*_*a*_	6	3 + *ε*	6	3 + *ε*
$\overset{¯}{c}$	2 − *ε*	5 + 2*ε*	5 + 3*ε*	10 − *ε*
Round 2 of *P*_*b*_	3*ε*	2	5	5 + 4*ε*
$\overset{¯}{c}$	2 − 4*ε*	3 + 2*ε*	3*ε*	5 − 5*ε*
Round 3 of *P*_*a*_	–	3 + *ε*	0	3 + *ε*
Round 3 of *P*_*b*_	–	–	3*ε*	–

*A*_*LO*_	12	12 + 2*ε*	12	12 + 2*ε*
*B*_*LO*_	5 + 7*ε*	7	7 + 3*ε*	5 + 7*ε*

## Footnotes

1 Surprisingly, the investigation of this problem from a game-theoretic perspective allowed to establish new results on the approximation ratio of a heuristic algorithm for the classical bin packing problem (Epstein, Kleiman, & Mestre, 2009).

2 In game theory, a strategy would denote the set of all decisions for any possible scenario of the game. This would be the result of a strategy in our more algorithmic terminology.
